# Adipocytokines in obese Ghanaian subjects with or without type 2 diabetes

**DOI:** 10.1186/s13104-018-3149-4

**Published:** 2018-02-08

**Authors:** Yussif Adams, Emmanuel Kwaku Ofori, Henry Asare-Anane, Seth D. Amanquah, Grace Korkor Ababio, Emmanuel Abendau, Richard Nabia

**Affiliations:** 1 Department of Biomedical and Laboratory Sciences, University of Development Studies, Tamale, Ghana; 2 Department of Physiology, University of Lausanne, Lausanne, Switzerland; 3 Department of Chemical Pathology, School of Biomedical and Allied Health Sciences (S.B.A.H.S), University of Ghana, P.O. Box 4236, Korle-Bu, Accra, Ghana; 4 Department of Biochemistry, S.B.A.H.S, Korle-Bu, Accra, Ghana; 5 Department of Physiology, S.B.A.H.S, Korle-Bu, Accra, Ghana; 6 Department of Nutrition and Dietetics, S.B.A.H.S, Korle-Bu, Accra, Ghana

**Keywords:** Leptin, Obesity, C-reactive protein, Type 2 diabetes

## Abstract

**Objective:**

This study aimed to evaluate serum leptin and high sensitivity C-reactive protein (hsCRP) concentrations in obese Ghanaians with or without type 2 diabetes and to find out the extent to which their levels are influenced by underlying disorders.

**Results:**

Obese subjects with type 2 diabetes had lower leptin but higher hsCRP levels compared with obese non-diabetic controls. There were negative correlations within the control group for glucose vs % muscle mass (r = − 0.378, p = 0.016), leptin vs % muscle mass (r = − 0.555, p = 0.001) and within the obese diabetic group for leptin vs % muscle mass (r = − 0.602, p = 0.001). Obese persons without diabetes were about three times more likely to have higher leptin levels compared with their obese diabetic counterparts (Odds ratio = 3.315, p < 0.001). Obese females independently had a tenfold increase in leptin levels compared with obese males.

**Electronic supplementary material:**

The online version of this article (10.1186/s13104-018-3149-4) contains supplementary material, which is available to authorized users.

## Introduction

Chronic disorders such as obesity and diabetes mellitus have reached epidemic proportions globally [1–3] with approximately eighty percent (80%) of the populace with type 2 diabetes mellitus either obese or overweight [4, 5]. Reports by International Diabetes Federation in 2015 revealed that an estimated (3.3–6.0) % of Ghanaians aged 20–79 years had diabetes mellitus with a further 7.8% being glucose impaired [1]. Inflammatory markers have generally been implicated in insulin resistance [6–9]. Leptin, a 16 kDa protein, secreted by the adipose tissue is a potential determinant of adiposity and risk for type 2 diabetes [10–13]. High sensitivity C-reactive protein (hsCRP), an acute phase protein is synthesized by the liver and increases in concentration following infection, inflammation or trauma [9]. Levels of hsCRP have been observed to be increased in obese persons with diabetes mellitus and correlate with measures of adiposity including body mass index (BMI) and waist circumference [14, 15]. The extent to which these biomarkers contribute to metabolic function and/or dysfunction is not fully understood especially in relation to gender and ethnicity. The purpose of this study was to evaluate serum leptin and hsCRP concentrations in Ghanaian obese subjects with and/or without type 2 diabetes and to find out the extent to which their levels are influenced by the underlying disorder. We hypothesized that obese subjects with type 2 diabetes will have higher leptin and hsCRP levels compared with their obese non-diabetic counterparts.

## Main text

### Methods

The study design was a cross-sectional one conducted among 160 obese (BMI > 30 kg/m^2^) Ghanaian subjects between October 2014 and April 2015. Study participants included 80 type 2 diabetic persons attending the National Diabetes Management and Research Centre (NDMRC), Korle-Bu, Accra and 80 age and gender-matched obese staff/workers of the Korle-Bu Teaching Hospital, Accra, Ghana without diabetes mellitus. Consecutive subjects who agreed to the study and met the criteria for inclusion were recruited. An oral glucose tolerance test (OGTT), regarded as diagnostic of type 2 diabetes were performed on all volunteers. Obesity was defined based on BMI ≥ 30 kg/m^2^. Type 2 diabetes was confirmed by a physician at the National Diabetes Management and Research Centre (NDMRC), Korle-Bu, Accra, based on results of fasting blood glucose ≤ 6.9 mmol/L and a 2 h-OGTT > 11.1 mmol/L on two separate occasions. Case subjects were either being lifestyle managed or were on oral hypoglycemic drugs. A pre tested structured (Additional file 1: Questionnaire) was administered to assess the socio-economic status, medical history and medications, family history of diabetes mellitus, and level of physical activity of subjects. Habitual smokers, defined as subjects who smoked tobacco or other smoking products (and are still smoking) continuously for at least 6 months, persons with gestational diabetes, chronic illnesses (having a persistent ailment for more than 3 months), stroke or amputation were excluded from the study. Assuming an odds ratio of 2 among obese subjects for type 2 diabetes, at 95% confidence interval and a power of 80%, a sample size of 60 persons were adequate for this study. The study was approved (Protocol Identification Number: MS-Et/M.6–P3.2/2014-2015) by the Institutional Ethics and Protocol Review Committee of School of Medicine and Dentistry, College of Health Sciences, University of Ghana. Detailed explanations on purpose of the study, risk and benefits were made known to participants. Written informed consent was obtained from all participants. Height of all participants were measured using a stadiometer (Secca, Germany). Weight was measured using a Full Body Sensor Body Composition Monitor and Scale (Omron model HBF-516, Omron Healthcare, USA). This scale employed bio-impedance analysis to compute percentage body fat (% body fat), percentage muscle mass (% muscle mass) and visceral fats. Blood pressure was taken using a mercury sphygmomanometer and stethoscope after participants had rested for 15 min. Venous blood (5 mL) was obtained from the subjects between 07:00 and 09:00 h each day, after an overnight fast, according to Helsinki protocol declaration (2008). One milliliter (1 mL) of whole blood was transferred into sodium fluoride containing tube and the plasma separated for the estimation of glucose. The remaining four milliliters (4 mL) of whole blood was placed into serum separator tubes for processing. Resulting sera were then aliquoted in 0.5 mL portions into sterile eppendorf tubes and stored at − 20 °C until required for use. Fasting blood glucose, total cholesterol (TCHOL), triglycerides (TG), low density lipoprotein (LDL) and high density lipoprotein cholesterol (HDL) were analyzed using auto-analyzer (Roche-Hitachi Modular Analytics, Tokyo, Japan) at the Department of Chemical Pathology Laboratory of the School of Biomedical and Allied Health Sciences, University of Ghana. Serum leptin and hsCRP levels were measured using enzyme-linked immunosorbent assay kits (GenWay Biotech Inc. VA, USA) at the Public Health Reference Laboratory, Accra. A calibration curve was used to determine analyte concentrations from the strength of signal produced in the immunoassay. Unspecific substrate binding was eliminated by using a specific secondary antibody. The GraphPad prism software version 6.0 (San Diego California, USA) was used for statistical analysis. Values are expressed as mean ± standard deviation. The unpaired student *t* test was used for the comparison between means of clinical and biochemical parameters. Several associations were tested using Pearson’s correlation co-efficient. The odds ratio with 95% confidence interval (CI) was calculated for all variables to determine their contribution to the variances in leptin levels. A probability level less than 5% was considered statistically significant.

## Results

Data were obtained from 80 persons with type 2 diabetes (cases) and 80 persons without diabetes mellitus (controls). The clinical and biochemical parameters of the study population are shown in Table 1. Difference in means for age, BMI, % body fat, % muscle mass, and visceral fat, respectively between the two groups were not significant (p = 0.9119, 0.2666, 0.4008, 0.1735, 0.4810). Case subjects had higher systolic blood pressure (SBP), glucose, hsCRP and lower TCHOL, LDL and leptin levels, respectively (p = 0.0001, 0.0009, 0.0036; 0.0135, 0.0069, 0.0018) than their control counterparts. When categorized into severity in glucose levels, male diabetic persons with glucose levels > 11.0 mmol/L and between 6.2 and 11.0 mmol/L had higher leptin and hsCRP levels, respectively (p = 0.0465; p = 0.0203) compared to the other grouping (Table 1). Associations between several correlates with body composition parameters are shown in Table 2. There were negative correlation within the obese non-diabetic group for glucose vs % muscle mass (r = − 0.378, p = 0.016), leptin vs % muscle mass (r = − 0.555, p = 0.001) and within the obese diabetic group for leptin vs % muscle mass (r = − 0.602, p = 0.001). Positive associations were observed for glucose vs % body fat (r = 0.315, p = 0.048), % body fat vs leptin (r = 0.572, p = 0.001), BMI vs leptin (r = 0.444, p = 0.004) within the control group and leptin vs % body fat (r = 0.635, p = 0.001) within the case group. Risk assessment of leptin levels in this study are provided in Table 3. Obese persons with diabetes were about three times more likely to have lower leptin levels than their obese non-diabetic counterparts (Odds ratio = 3.315, CI = 1.918, 5.824; p < 0.001). Further, obese women, independent of other factors were about 10 times more likely to have higher leptin levels than obese men (Odds ratio = 9.750, CI = 4.461, 21.311; p < 0.001).

**Table 1:** Clinical and biochemical measurements of study participants

Variables	Obese subjects		p value
	Diabetics (80)	Non-diabetics (80)	
Age	48.60 ± 5.928	48.40 ± 7.877	0.9119
Sex (M/F)	30/50	30/50	
Height (cm)	172.2 ± 9.996	168.6 ± 4.623	0.0734
Weight (kg)	98.55 ± 18.53	91.30 ± 8.952	0.0586
BMI (kg/m^2^)	34.08 ± 2.97	33.13 ± 2.361	0.2666
% Body fat	30.19 ± 7.319	31.47 ± 3.969	0.4008
% Muscle mass	32.72 ± 3.009	31.64 ± 3.062	0.1735
Visceral fat	16.00 ± 4.202	15.27 ± 3.796	0.4810
SBP (mmHg)	138.4 ± 9.027	126.9 ± 7.780	*0.0001*
DBP (mmHg)	85.87 ± 8.295	83.53 ± 6.684	0.2351
Glucose (mmol/L)	6.500 ± 2.956	4.947 ± 0.739	*0.0009*
TCHOL (mmol/L)	4.369 ± 0.898	5.059 ± 1.181	*0.0135*
TG (mmol/L)	1.515 ± 1.206	1.366 ± 0.745	0.5678
HDL (mmol/L)	1.019 ± 0.202	1.021 ± 0.275	0.9830
LDL (mmol/L)	2.724 ± 0.744	3.345 ± 0.959	*0.0069*
CR (ratio)	4.437 ± 1.259	5.158 ± 1.399	*0.0402*
Leptin (ng/ml)	13.84 ± 4.759	17.92 ± 5.506	*0.0018*
hsCRP (ng/ml)	0.256 ± 0.108	0.184 ± 0.106	*0.0036*

Variables	Glucose level among diabetic subjects			p value
	3.6–6.19 mmol/L	6.2–11.0 mmol/L	> 11.0 mmol/L	
Male				
Leptin	10.33 ± 4.08^b^	10.14 ± 1.88^b^	6.000 ± 0.71^a^	*0.0465*
hsCRP	0.193 ± 0.09^b^	0.314 ± 0.12^a^	0.150 ± 0.02^b^	*0.0203*
Female				
Leptin	14.60 ± 3.19	15.95 ± 3.86	15.83 ± 2.021	0.3484
hsCRP	0.301 ± 0.10	0.264 ± 0.13	0.202 ± 0.138	0.4913

Values are given as mean ± standard deviation

Categorized glucose grouping by one way ANOVA

Italic results indicate significant relationships

*M* male; *F* is female; *SBP* systolic blood pressure; *DBP* diastolic blood pressure; *TCHOL* total cholesterol; *TG* triglyceride; *HDL* high density lipoprotein; *LDL* low density lipoprotein; *BMI* body mass index; *hsCRP* high sensitivity C-reactive protein; *CR* coronary risk; *%* percentage

^a, b^Denotes differences between groups. p < 0.05 is statistically significant

**Table 2:** Association between biochemical indices and body composition parameters

Variables	Control group (obese non-diabetics (80)				Case group (obese diabetics (80)			
	BMI	Body fat	Muscle mass	Visceral fat	BMI	Body fat	Muscle mass	Visceral fat
Glucose								
r	0.258	*0.315*	**−** *0.378*	-0.091	0.077	0.104	− 0.080	0.145
p	0.107	*0.048*	*0.016*	0.579	0.636	0.525	0.624	0.373
TCHOL								
r	− 0.004	− 0.138	0.195	0.300	0.078	0.004	0.022	− 0.077
p	0.981	0.397	0.229	0.060	0.633	0.979	0.895	0.638
TG								
r	0.025	− 0.301	*0.351*	*0.504*	0.075	− 0.178	0.197	0.222
p	0.878	0.059	*0.026*	*0.001*	0.644	0.271	0.224	0.169
HDL								
r	− 0.227	0.165	− 0.160	− 0.179	− 0.033	0.117	− 0.101	− 0.230
p	0.159	0.310	0.325	0.357	0.841	0.473	0.535	0.153
LDL								
r	0.131	− 0.057	0.125	0.292	0.075	0.024	− 0.004	− 0.090
p	0.421	0.727	0.443	0.067	0.645	0.884	0.978	0.579
VLDL								
r	0.029	− 0.303	*0.355*	*0.515*	0.076	− 0.184	0.204	0.222
p	0.858	0.058	*0.025*	*0.001*	0.641	0.255	0.207	0.169
CR								
r	0.278	− 0.166	0.242	*0.350*	0.044	− 0.070	0.062	0.076
p	0.082	0.306	0.133	*0.027*	0.786	0.668	0.705	0.639
Leptin								
r	*0.444*	*0.572*	**−** *0.555*	− 0.114	0.237	*0.635*	**−** *0.602*	− 0.144
p	*0.004*	*0.001*	*0.001*	0.483	0.141	*0.001*	*0.001*	0.377
hsCRP								
r	0.033	0.211	− 0.158	− 0.005	0.172	0.113	− 0.035	0.125
p	0.840	0.191	0.329	0.973	0.289	0.489	0.830	0.443

Table 2 shows the association between several correlates with body composition

Italic results indicate significant relationships

*TCHOL* total cholesterol; *HDL* high density lipoprotein; *LDL* low density lipoprotein; *VLDL* very low density lipoprotein; *TG* triglyceride; *BMI* body mass index; *hsCRP* high sensitivity C-reactive protein; *CR* coronary risk

**Table 3:** Risk assessment of serum leptin in study participants

Variable	OR	p-value	95% CI	aOR	p-value	95% CI
Diabetics						
Non-diabetics	*3.315*	*<* *0.0001*	1.918–5.824	*3.217*	*<* *0.0001*	1.008–6.045
Gender						
Male						
Female	*9.750*	*<* *0.0001*	4.461–21.311	*9.561*	*<* *0.0001*	4.060–24.200
Exercise						
Yes						
No	1.296	0.466	0.646–2.600	1.245	0.566	0.588–2.636
Viceral fats						
< 9.0						
> 9.0	0.163	0.090	0.020–1.330	0.223	0.176	0.025–1.962
Family history of DM						
No						
Yes	0.655	0.211	0.338–1.270	1.018	0.962	0.490–2.114
TCHOL						
3.5–6.5 mmol/L						
> 6.5 mmol/l	1.453	0.671	0.259–8.157	0.819	0.83	0.132–5.094
TG						
1.7–2.2 mmol/L						
> 2.2 mmol/l	0.711	0.737	0.098–5.163	1.041	0.97	0.128–6.477
HDL						
1.3–1.5 mmol/L						
> 1.5 mmol/l	1.835	0.476	0.346–3.739	1.764	0.532	0.298–4.434
LDL						
3.37–4.12 mmol/L						
> 4.12 mmol/l	0.685	0.428	0.269–1.746	0.418	0.099	0.148–1.179

Risk assessment for serum leptin levels within the study population

Italic results indicate significant relationships

*CI* confidence interval; *OR* odd ratio; *aOR* adjusted odd ratio; *TCHOL* total cholesterol; *TG* triglyceride; *HDL* high density lipoprotein; *LDL* low density lipoprotein

## Discussion

Obesity is a major contributor to the burden of metabolic dysfunction and to several chronic diseases including type 2 diabetes [1]. In the southern parts of Ghana, especially in the Greater Accra region and its environs, the prevalence of obesity and type 2 diabetes have been reported to be increasing steadily [2, 16]. What is not clear is the contributory effects of gender, physical activity and diabetes mellitus on obesity. The present study evaluated the impact of serum leptin and hsCRP levels in obese Ghanaian subjects with and/or without type 2 diabetes. In this study, obese subjects with type 2 diabetes had lower serum leptin but higher hsCRP concentrations than obese non-diabetic individuals. Multivariate analysis further revealed that, obese persons with type 2 diabetes were three times less likely to have elevated leptin levels than obese controls. Further, obese diabetic men in this study, with poor glycemic control, had lower leptin levels. A possible explanation for reduced leptin levels in persons with type 2 diabetes may be due to insulin resistance, a modulator of leptin production. Thus defect in glucose sensing, as seen in type 2 diabetes could be responsible for the lower leptin production, suggesting a signalling crosstalk in glucose regulation. Altered body fat distribution in diabetic subjects have been suggested as an alternative explanation for lowered leptin levels [17–20]. Even though this study did not observe differences in visceral fat among the two groups, leptin levels correlated positively with measures of adiposity and body composition in both groups indicating a possible synergistic effect. A third explanation could be due to the use of statin medications. Twelve (12) of our case subjects were on lipid lowering drugs and this could have contributed to such an observation. Result however were not statistically significantly when we excluded those on lipid lowering drugs from the analysis. Serum leptin levels in this study was also influenced by gender. Obese females were about 10 times more likely to have higher leptin levels than obese males. Stimulation of leptin mRNA production by 17β-estradiol in females could explain the above observation [21]. An inverse relationship has also been observed in a male cohort for leptin versus testosterone levels [22]. Levels of hsCRP among obese persons with type 2 diabetes in this study, was significantly higher than controls suggesting a possible directional relationship between insulin resistance and chronic inflammation. Whereas this study showed no variations in body composition, prior studies showed a strong association between hsCRP and measures of central obesity [23]. In summary, results highlight relationships of leptin and hsCRP with obesity and type 2 diabetes. We demonstrated that type 2 diabetes and gender were independent factors in determining serum leptin levels among obese subjects.

## Limitations

This study had some limitations. Although the authors primarily focused on obese subjects, a normal weight control group would have been useful in a cross-sectional comparison manner. Another limitation was our inability to measure the insulin resistant state of our subjects. Further, the authors could not measure glycated hemoglobin (HbA1c), a marker that could have provided information on diabetes management. Future studies should focus on large population-based longitudinal studies to understand with change over time, the mechanistic role of adipocytokines in obesity and related disorders.

## Additional file

**Additional file 1: Questionnarie.** Study questionnaire. This file contains the pre-tested questionnaire used in the study.

## Abbreviations

BMI: body mass index
hs-CRP: high sensitivity C-reactive protein
OGTT: oral glucose tolerance test
SBP: systolic blood pressure
DBP: diastolic blood pressure
TG: triglyceride
TCHOL: total cholesterol
LDL: low density lipoprotein
HDL: high density lipoprotein
CR: coronary risk
HbA1c: glycated hemoglobin
OR: odds ratio
CI: confidence interval
