# Supplementary material for: Adipocytokines in obese Ghanaian subjects with or without type 2 diabetes
Source: BMC Res Notes. 2018 Feb 8;11:109. doi: 10.1186/s13104-018-3149-4 (PMC5806467; doi:10.1186/s13104-018-3149-4)
Supplement: Supplementary file 1 — Additional file 1: Questionnarie. Study questionnaire. This file contains the pre-tested questionnaire used in the study. [file 13104_2018_3149_MOESM1_ESM.docx]

**QUESTIONNAIRE**

Name: ……………………………………………. Code: ……………………………..

Date: …………………………………… Patient ID: ………………………………….

Tel.: …………………………............. E-mail: …………….……………………….....

Postal Address: …………………………………………………………………………

**Demographic and Anthropometric measurement**

1. Age: ………………………. 6. Height (m): ……………………….
2. Weight (kg): ……………… 7. BMI (kg/m^2^): ……………..………
3. BP: ……………………….. 8. Pulse: ……………………………..
4. % body fat: ………………. 9. Visceral fat: ………………………
5. % Muscle mass: ………….. 10. Bone mass: ………………..………

**Please tick [ ] the appropriate box where applicable**

1. Sex [ ] male

[ ] female

1. Marital status [ ] single [ ] married

[ ] divorced [ ] widowed

1. Education [ ] none [ ] primary

[ ] secondary [ ] tertiary

1. Occupation [ ] unemployed [ ] trader/self employed

[ ] government worker [ ] others (please specify)……

1. Ethnicity [ ] Akan [ ] Ga [ ] Ewe

[ ] Northerner [ ] others (please specify)……

**Lifestyle**

1. How many times do you eat in a day?

[ ] two

[ ] three

[ ] four

[ ] five

1. What type of food do you mostly eat?

[ ] more carbohydrates and less meat or fish

[ ] less carbohydrates and more vegetables and fruits

[ ] less carbohydrates and more meat and fish

1. Do you exercise?

[ ] yes

[ ] no

If yes to question 13, how many times do you exercise?

[ ] once a week

[ ] twice a week

[ ] 3 – 5 times a week

[ ] every day of the week

**Medical History**

1. Do you smoke cigarette?

[ ] yes

[ ] no

If yes, [ ] 1 pack/day [ ] 2 pack/day [ ] >2 pack/day

1. Family history of diabetes mellitus

[ ] yes

[ ] no

1. Do you have any of the following conditions?

[ ] none [ ] hypertension [ ] diabetes mellitus

[ ] chronic kidney disease [ ] others (please specify)…

1. Are you taking any herbal remedy or preparation? [ ] yes

[ ] no

**Clinical data**

1. Duration of diabetes since diagnosis

[ ] < 6months [ ] 1 year [ ] 2 years [ ] 5 years [ ] >5years

1. Do you experience any of the following complications

[ ] none [ ] retinopathy [ ] neuropathy

[ ] nephropathy [ ] skin lesion [ ] recurrent infection

[ ] cardiovascular disease [ ] oral cavity lesion

[ ] others (please specify)………………………

1. Type of drugs: [ ] none [ ] metformin [ ] rosiglitazone [ ] insulin [ ] Angiotensin receptor blockers (ARB)

[ ] Angiotensin-converting enzymes (ACE) inhibitors

[ ] beta blockers [ ] others (please specify)…….………

1. Are you taking any herbal remedy or preparation?

[ ] yes

[ ] no

.

**Thank you for participating in this study**
